# Prevalence and Molecular Characterization of *Campylobacter* spp. Isolated from Patients with Diarrhea in Shunyi, Beijing

**DOI:** 10.3389/fmicb.2018.00052

**Published:** 2018-01-26

**Authors:** Ying Li, Shuang Zhang, Mu He, Yanchun Zhang, Yanyan Fu, Hao Liang, Hongbo Jing, Yindong Li, Hongmei Ma, Maojun Zhang

**Affiliations:** ^1^Shunyi District Center for Disease Control and Prevention, Beijing, China; ^2^National Institute for Communicable Disease Control and Prevention, Beijing, China

**Keywords:** *Campylobacter* infection, adult diarrheal patients, prevalence, molecular subtyping, antimicrobial susceptibility

## Abstract

Bacterial pathogens have been confirmed as the major cause of acute diarrhea among outpatients in China. In this study, 370 stool samples from the patients aged from 15 to 87 years old with diarrhea were collected over 12 months (from May 2016 to April 2017) in two hospitals in Shunyi, Beijing. Bacterial isolation was performed for the common enteric pathogens: *Campylobacter, Salmonella, Shigella, Diarrheagenic Escherichia coli*, and *Vibrio parahaemolyticus* for 370 samples. The filtration method was used for the *Campylobacter* isolation in this study. The prevalence and molecular characterization of the *Campylobacter* were investigated. The isolation ratio for *Campylobacter*, *Salmonella*, *Shigella*, *Diarrheagenic E. coli,* and *V. parahaemolyticus* was 7.0% (26/370), 6.2% (23/370), 0.3% (1/370), 7.3% (27/370), and 10.3% (38/370), respectively. Based on the isolation result, *Campylobacter* positive cases presented in almost every month of the whole year and the isolation ratio was the highest among the tested pathogens during October to March. There was no significant difference between genders of *Campylobacter* positive cases. More *Campylobacter* positive cases presented dehydration compared with those who were positive for *Salmonella*. Twenty-six *Campylobacter* isolates were obtained in this study and 24 of these were *Campylobacter jejuni.* The antibiotic susceptibility tests indicated that 83.3% (20/24) of the isolates exhibited resistance to three or more types of antibiotic. Twenty STs were identified for the 26 *Campylobacter* isolates and four novel STs were identified in this study. No clonal cluster was found among these isolates. This is the first study for *Campylobacter* isolated using the filtration method in China which indicated the *Campylobacter* infection might be seriously under-ascertained in the diarrheal patients in China.

## Introduction

Diarrheal disease remains an important medical conundrum and causes significant morbidity and mortality in developing countries (Allos, 2001; Guerrant et al., 2001; Samosornsuk et al., 2015; Nohra et al., 2016; Hatanaka et al., 2017; Ramonaite et al., 2017; Wozniak-Biel et al., 2017). *Campylobacter*, *Salmonella*, *Shigella*, *Diarrheagenic E. coli* (DEC), and *Vibrio parahaemolyticus* are the common pathogens for diarrhea. *Campylobacter* is a food-borne zoonotic pathogen, and is considered to be one of the most common causes of bacterial gastroenteritis both in developed and developing countries. From the 1980s to 2000, numerous studies investigated *Campylobacter* infection in diarrheal patients in China and the isolation ratio reached to 10–20% (Young et al., 1986; Desheng et al., 1992; Chen et al., 1995). However, after 2000, fewer reports about the prevalence and characteristics of the *Campylobacter* infection in the diarrheal patients, and the isolation ratio decreased significantly (Wang et al., 2015).

The laboratory diagnosis of bacterial infection is mainly based on traditional culture methods (Wei et al., 2014; Wang et al., 2015; Zhang et al., 2017). Currently, conventional *Campylobacter* isolation method from the stool sample is based on the direct plating the samples on the selective medium (Yoo et al., 2014). One hypothesis suggests that the increasing drug resistance in bacteria in the samples significantly affects the selective culture methods, especially if there is multi-drug resistant *Proteus mirabilis* contamination in the sample. It is not yet known why there is such a low detection rate and whether this is a true decrease in campylobacteriosis in recent years in China or just because of the invalid conventional *Campylobacter* isolation methods used.

In this study, the detection of *Campylobacter* with a novel laboratory modified *Campylobacter* isolation kit based on the filtration method was used on stool samples collected from diarrheal patients visiting two community clinics in Shunyi district, Beijing. The prevalence study was performed over 1 year. Antibiotic susceptibility tests and molecular sub-typing analysis were performed for all of the *Campylobacter* isolates. Simultaneously, the isolation for other four pathogenic organisms, *Salmonella, Shigella, V. parahaemolyticus,* and DEC which including the enteropathogenic *Escherichia coli* (EPEC), enterotoxigenic *E. coli* (ETEC), enterohemorrhagic *E. coli* (EHEC), enteroinvasive *E. coli* (EIEC) and enteroaggregative *E. coli* (EAEC) were also investigated for the samples in this study. We conducted one general practice-based investigations for the major enteric pathogens in diarrheal patients visiting community hospitals in this study.

## Materials and Methods

### Sample Collection

According to the guidelines of the local foodborne disease surveillance project in Beijing, diarrheal patients enrolled in this study were outpatients who presented with acute diarrhea. This was defined as ≥3 passages of watery, loose, mucus-, or bloody-stools during a 24 h period in two clinics (Shunyi and Konggang Hospitals) in Shunyi district, Beijing from May 1, 2016 to April 30, 2017 inclusive. Five milligrams of fresh stool samples were collected from each of the diarrheal patients. The samples were stocked in Cary-Blair medium at 4°C and transported to the laboratory for bacterial isolation within 24 h. Totally, 370 stool samples from 370 cases were collected (251 samples from Shunyi Hospital and 119 samples from Konggang Hospital). Among the 370 cases, 225 were male and 145 were female. The age of the patients ranged from 15 years old to 87 years old. The bacterial isolation for *Campylobacter, Salmonella, Shigella, V. parahaemolyticus,* and DEC was performed for all collected samples. The prevalence characteristics of the *Campylobacter* infection were analyzed according to the questionnaire information from each of the individual patient inquired by the clinical doctor during hospital visiting time for each patient (Supplementary File S1).

### Isolation of *Campylobacter, Salmonella, Shigella,* DEC, and *Vibrio parahaemolyticus*

*Campylobacter* spp. isolation was carried out using the *Campylobacter* isolation kit incorporating a membrane filter method (ZC-CAMPY-002, Qingdao Sinova Biotechnology Co., Ltd., Qingdao, China). Briefly, 1 mL stool specimen suspension was transferred into 4 mL enrichment buffer which was provided in the kit. The principle component of the enrichment buffer was the modified Preston broth which was described in the manual book. The enriched suspension was incubated at 42°C for 24 h in a microaerophilic atmosphere consisting of 5% O_2_, 10% CO_2_, and 85% N_2_. Three hundred μL cultured enrichment suspension was then spotted on to the surface of the filter pasted on to the double medium plates, which contained Karmali and Columbia agar, respectively. The medium plates were incubated in a microaerophilic atmosphere at 42°C for 48 h. The suspected colonies were picked and identified by Gram stain and biochemical tests. Multiple PCR tests were used to confirm and identify the species according to the previous study (Wang et al., 2002).

Conventional culture methods were used for *Salmonella, Shigella,* DEC, and *V. parahaemolyticus*. One mL stool sample was inoculated into 5 mL selenite brilliant green broth and enriched at 36 ± 1°C for 18–24 h (Wang et al., 2015). After selective enrichment, a loop of the culture was streaked on Chromagar and incubated at 36 ± 1°C for 18–24 h. More than one presumptive *Salmonella* colony (usually 3–5 colonies) on the selective agar plate were inoculated on to triple sugar iron slant and incubated at 36 ± 1°C for 24 h. Isolates with typical *Salmonella* phenotypes were confirmed by systemic chemical tests followed by serotyping with serological test (*Salmonella* antisera, SSI, Denmark) (Brenner et al., 2000).

One loop of the stool sample was directly inoculated on to xylose lysine desoxycholate (XLD) medium and MacConkey (MAC) plates and cultured at 36°C for 24 h. Two non-fermented lactose colonies and four lactose-fermenting colonies were picked from the MAC plates. The colorless and transparent colonies were picked from the XLD plates. For each selected colony, the pure culture was amplified using the Tryptose Soya agar medium for 24 h. The pure culture for each suspected colony was identified using VITEK^®^ 2 COMPACT (BioMerieux) based on the biochemical test. The isolates identified as *Shigella* were further identified by serological typing (Japanese Health Research Serum). Six of the suspected colonies were further identified using real-time PCR according to previous study for *eaeA*, *stx_1_* and *stx_2_* genes of EHEC; *eaeA* and *escV* of EPEC; *ipaH* of EIEC; *lt, sth,* and *stp* of ETEC; and *aggR* of EAEC. The PCR conditions were as follows: initial denaturation at 95°C for 5 min, followed by 45 cycles of 95°C for 15 s and 60°C for 1 min. A positive reaction was identified as the *C*t value was less than 35 (Chen et al., 2014).

Simultaneously, another loop of stool sample was directly inoculated into 5 mL 3% Sodium Chloride Alkaline Peptone Water and enriched at 36°C for 24 h. Then, one loopful of enriched culture was streaked on to Chromagar and incubated at 36°C for 24 h. Three to five colonies of suspected *V. parahaemolyticus* colonies on the selective agar plate were transferred onto triple sugar iron slant and incubated at 36°C for 24 h. Isolates with typical *V. parahaemolyticus* phenotypes were confirmed by systemic chemical tests followed by serotyping with a commercial kit (*V. parahaemolyticus* antisera, Denka Seiken, Japan).

### Antimicrobial Susceptibility Testing for *Campylobacter*

The Minimum Inhibitory Concentrations (MICs) for 11 antimicrobials (erythromycin, azithromycin, nalidixic acid, ciprofloxacin, gentamicin, streptomycin, chloramphenicol, florfenicol, tetracycline, telithromycin, and clindamycin) were determined using an agar dilution method as recommended by CLSI (M100-S25, 2015). Briefly, Mueller–Hinton agar (CM0337, Oxoid, United Kingdom) plates supplemented with 5% defibrinated sheep blood and double diluted antimicrobial agents at concentrations ranging from 0.02 to 256 μg ml^-1^ were used. The turbidity of the bacterial suspension was 0.5 McFarland (approximately 10^8^ CFU ml^-1^). Plates were inoculated with a multipoint inoculation instrument with 1 mm diameter inoculating pins and incubated at 37°C for 48 h under microaerobic conditions. Inoculated plates started with the lowest concentration. The growth control plates were inoculated before and after each inoculation for different agents. The MIC was read as the lowest concentration without visible growth.

The breakpoints for resistance used in this study were those with a MIC value greater than or equal to the values according to the standard used in the National Antimicrobial Resistance Monitoring System (NARMS-2014) for *Campylobacter* in the United States: erythromycin (≥32 μg mL^-1^), azithromycin (≥8 μg mL^-1^), nalidixic acid (≥64 μg mL^-1^), ciprofloxacin (≥4 μg mL^-1^), gentamicin (≥8 μg mL^-1^), streptomycin (≥16 μg mL^-1^), chloramphenicol (≥32 μg mL^-1^), florfenicol (≥8 μg mL^-1^), tetracycline (≥16 μg/mL^-1^), telithromycin (≥16 μg mL^-1^), and clindamycin (≥8 μg mL^-1^). *C. jejuni* ATCC 33560, *Staphylococcus aureus* ATCC 29213, and *E. coli* ATCC 25922 were used as controls.

### Multilocus Sequence Typing for *Campylobacter*

Multilocus sequence typing (MLST) was performed by amplifying and sequencing seven housekeeping genes loci, *aspA, glnA, gltA, glyA, pgm, tkt*, and *uncA*, and by using previously described primers for *C. jejuni* and *C. coli*^1^. The nucleotide sequences of the amplicons were determined using the published oligonucleotide primers and reaction conditions (Dingle et al., 2001). Allele numbers and STs were assigned using the *Campylobacter* MLST database^2^.

### Statistical Analysis

The entire statistical analysis was performed using Stata software, version 12.0. The Chi-square test was used for the comparisons for the isolation ratios of different pathogens in different month. The *Campylobacter* infection ratio between different gender, among different age group and contamination in different suspected food groups were also analyzed using Chi-square test. Statistical significance was set at *P*-value < 0.05. Two-sided test was used.

### Ethics Statement

All aspects of the study were performed in accordance with national ethics regulations and approved by the Ethics Committee of the Shunyi Hospital and Konggang Hospital as well as Shunyi District CDC, China. Participants received information on the study’s purpose and of their right to keep information confidential. Written consent was obtained from each participant and children’s parents or their guardians.

## Results

### Epidemiological Information and Pathogens Spectrum

A total of 370 diarrheal patients (225 male and 145 female) were enrolled from the local population over the course of an entire year. Patients ranged in age from 15 to 87 years old. 370 stool samples were collected from 370 diarrheal cases. There were 108 (29.2%, 108/370) cases that were positive for the tested enteric pathogens. The prevalence for *Campylobacter*, non-typhoidal *Salmonella*, *Shigella*, DEC and *V. parahaemolyticus* was 7.0% (26/370), 6.2% (23/370), 0.3% (1/370), 7.3% (27/370), and 10.3% (38/370), respectively. The sero-typing results for the 23 non-typhoidal *Salmonella* isolates were 10 *S*. Typhimurium, 5 *S*. Enteritidis, 3 *S*. Agona, 1 *S*. Rissen, 1 *S*. Thompson, 1 *S*. Uganda, 1 *S*. Indiana, and 1 *S*. Stanleyville, respectively. In a total, seven (2.0%, 7/370) cases had co-infections with two different pathogens. Among these, six cases were co-infected with DEC and other pathogens (3 cases with *V. parahaemolyticus,* 2 cases with *Salmonella* and1 case with *Campylobacter*). Another case had *Salmonella* and *Campylobacter* co-infection. The isolation ratio and the samples collected from each month are listed in **Table 1**. It is interesting that 94.6% (35/37) of the *V. parahaemolyticus* infections presented from July to September, while 88.9% (24/27) of the DEC infections presented from June to October, and 82.6% (19/23) of the *Salmonella* infections presented between April and July. However, those with *Campylobacter* infections more or less presented in each month. From October to the following March, *Campylobacter* infection was isolated more frequently than other tested pathogens. After March, other pathogens increased significantly, especially *Salmonella* infection (**Table 1**).

**Table 1:** The isolation ratio of bacterial pathogens by month with samples collected from diarrheal patients.

Month	*Campylobacter %* (P/T)^a^	*Salmonella %* (P/T)	*Shigella %* (P/T)	*Diarrheagenic Escherichiacoli %* (P/T)	*Vibrio parahaemolyticus %* (P/T)
May 2016	1.8 (1/56)	5.4 (3/56)	0.0 (0/56)	1.8 (1/56)	3.6 (2/56)
June 2016	4.2 (2/48)	6.3 (3/48)	0.0 (0/48)	14.6 (7/48)	2.1 (1/48)
July 2016	8.5 (5/59)	6.8 (4/59)	0.0 (0/59)	13.6 (8/59)	22.0 (13/59)
August 2016	5.3 (2/38)	0.0 (0/38)	0.0 (0/38)	7.9 (3/38)	44.7 (17/38)
September 2016	2.5 (1/40)	2.5 (1/40)	0.0 (0/40)	10.0 (4/40)	12.5 (5/40)
October 2016	10.0 (4/40)	7.5 (3/40)	2.5 (1/40)	5.0 (2/40)	0.0 (0/40)
November 2016	18.2 (2/11)	0.0 (0/11)	0.0 (0/11)	0.0 (0/11)	0.0 (0/11)
December 2016	0.0 (0/9)	0.0 (0/9)	0.0 (0/9)	0.0 (0/9)	0.0 (0/9)
January 2017	25.0 (1/4)	0.0 (0/4)	0.0 (0/4)	0.0 (0/4)	0.0 (0/4)
February 2017	8.3 (1/12)	0.0 (0/12)	0.0 (0/12)	0.0 (0/12)	0.0 (0/12)
March 2017	10.0 (1/10)	0.0 (0/10)	0.0 (0/10)	10.0 (1/10)	0.0 (0/10)
April 2017	14.0 (6/43)	20.9 (9/43)	0.0 (0/43)	2.3 (1/43)	0.0 (0/43)
Total	7.0 (26/370)	6.2 (23/370)	0.3 (1/370)	7.3 (27/370)	10.3 (38/370)

^a^*P = Number of positive patients in each month; T = Number of total patients in each month*.

### Characteristics of *Campylobacter* Infection

Twenty six campylobacteriosis cases were observed in this study. Twenty-four (92.3%) cases were infected with *C. jejuni* and two (7.7%) cases were infected with *C. coli*. Among the 26 *Campylobacter* positive cases, one was a *C. coli* co-infected with *Salmonella*; another case was a *C. jejuni* co-infected with DEC. Among the 26 cases, 19 were male and 7 were female. However, there was no significant difference between infection ratio and gender (8.44% vs. 4.83%, *P* = 0.184, χ^2^ = 1.766). The age distribution of the *Campylobacter* and *Salmonella* positive ratio are presented in **Table 2**. According to the statistical analysis, there was no significant difference for the *Campylobacter* infection among different age group (*P* = 0.558, χ^2^ = 2.997), and the positive ratio between *Campylobacter* and non-typhoidal *Salmonella* had no difference among the age groups (**Table 2**).

**Table 2:** The age distribution of the *Campylobacter* and *Salmonella* positive cases.

Age group	*Campylobacter %*	*Salmonella %*	*P*-value	χ^2^
(year)	(P/T)^a^	(P/T)		
15–30	7.8 (11/142)	5.6 (8/142)	0.476	0.508
31–45	5.2 (6/115)	8.7 (10/115)	0.300	1.075
46–60	5.0 (3/60)	3.3 (2/60)	0.500^b^	–
61–75	10.0 (4/40)	2.5 (1/40)	0.179^b^	–
76–87	15.4 (2/13)	15.4 (2/13)	0.700^b^	–

^a^*P = Number of positive patients in each age group; T = Number of total patients in each age group. ^b^Fisher’s exact test*.

Each of the 26 cases had diarrhea more than 3 times per day and 12 of them had liquid stools, 7 of them had loose stools and the remaining 7 cases excreted stool shapes of a consistency between that of liquid and loose stools. No bloody stools were found among the 26 adult cases. The major clinical symptoms reported by the patients were abdominal pain (17 cases), nausea and vomiting (14 cases), dehydration (13 cases), feeling thirsty (9 cases), fever (8 cases), and feeling weak (7 cases). The number of the dehydration cases in the *Campylobacter* positive cases was higher than in the *Salmonella* positive cases (**Table 3**). The dehydration was determined by the physician that one of the syndrome appeared as decreased skin elasticity, dry skin, accelerated or weakened pulse, collapsed superficial vein, frosty limbs and decreased urine volume.

**Table 3:** Comparison of the clinical outcomes between *Campylobacter* and *Salmonella* infection.

Clinical symptoms	*Campylobacter* infection	*Salmonella* infection	*P*-value	χ^2^
Abdominal pain	17	12	0.348	0.882
Nausea and vomiting	14	16	0.49	0.477
Dehydration	13	4	0.036	4.379
Feeling thirsty	9	4	0.299	1.079
Fever	8	12	0.128	2.315
Feeling weak	7	4	0.649	0.207

The suspected contaminated foods were asked by the physicians according to the questionnaire for each patient. Each patient was asked to recall the diet in 5 days before the onset of the disease, and recounted the dubious food by himself. The entire suspected contaminated foods from the 370 cases were classified into nine groups: fruit and vegetables, meats and related products, grain and related products, water, beverages and herbs, seafood and related products, bean and related products, milk and related products, eggs and related products and unknown group. The positive ratio of *V. parahaemolyticus* was significantly higher in the seafood and related products group. There was no significant difference for the positive ratio of each pathogen in the other groups (**Table 4**).

**Table 4:** Positive ratio of the tested pathogens in the patients recalled different foods sources.

Food types	Positive rate of pathogens infection *%* (P/T)^a^				*P*-value	χ^2^
	*Campylobacter*	*Salmonella*	*Vibrio parahaemolyticus*	*Diarrheagenic E. coli*		
Fruit and vegetables	8.0 (10/126)	5.6 (7/126)	7.9 (10/126)	6.4 (8/126)	0.843	0.942
Meats and related products	7.0 (5/71)	8.5 (6/71)	17.0 (12/71)	8.5 (6/71)	0.193	4.724
Grain and related products	10.0 (5/50)	4.0 (2/50)	8.0 (4/50)	12.0 (6/50)	0.522	2.25
Water, Beverages and Herbs	12.8 (5/39)	7.7 (3/39)	7.7 (3/39)	7.7 (3/39)	0.815	0.942
Seafood and related products	0.0 (0/28)	0.0 (0/28)	21.4 (6/28)	7.1 (2/28)	0.005	12.923
Bean and related products	0.0 (0/9)	11.1 (1/9)	0.0 (0/9)	11.1 (1/9)	0.548	2.118
Milk and related products	0.0 (0/8)	12.5 (1/8)	12.5 (1/8)	0.0 (0/8)	0.545	2.133
Eggs and related products	0.0 (0/5)	0.0 (0/5)	40.0 (2/5)	20.0 (1/5)	0.23	4.314
Unknown	2.9 (1/34)	8.8 (3/34)	0.0 (0/34)	0.0 (0/34)	–	–

^a^*P = Number of positive patients recalled different foods sources; T = Number of total patients recalled different foods sources*.

### Antibiotic Susceptibility and Molecular Sub-typing of *Campylobacter*

**FIGURE 1:**
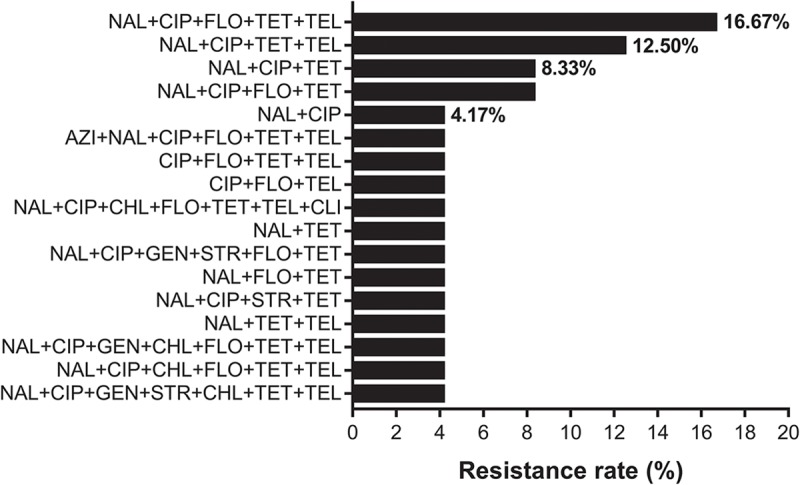
The resistance spectrum of 24 strains of *Campylobacter jejuni* to various antibiotic combinations. The *X*-axis represents the resistance rate of *Campylobacter*. The *Y*-axis represents a series of combination of antibiotics. Twenty (83.3%, 20/24) isolates were multi-drug resistant. Full and abbreviation name of antibiotics were: azithromycin (AZI), nalidixic acid (NAL), ciprofloxacin (CIP), gentamicin (GEN), streptomycin (STR), chloramphenicol (CHL), florfenicol (FLO), tetracycline (TET), telithromycin (TEL), and clindamycin (CLI).

Two *C. coli* isolates were sensitive only to florfenicol and chloramphenicol. They were resistant to the nine aforementioned antibiotics. All *C. jejuni* isolates were sensitive to erythromycin. The resistance ratio for *C. jejuni* was: tetracycline (91.7%), nalidixic acid (91.7%), ciprofloxacin (87.5%), telithromycin (62.5%), florfenicol (58.3%), chloramphenicol (16.7%), streptomycin (12.5%), gentamicin (12.5%), clindamycin (4.2%), and azithromycin (4.2%). Twenty (83.3%, 20/24) isolates were multi-drug resistant. The dominant resistance pattern was nalidixic acid, ciprofloxacin, florfenicol, tetracycline and telithromycin combined resistance (16.7%, **Figure 1**).

**FIGURE 2:**
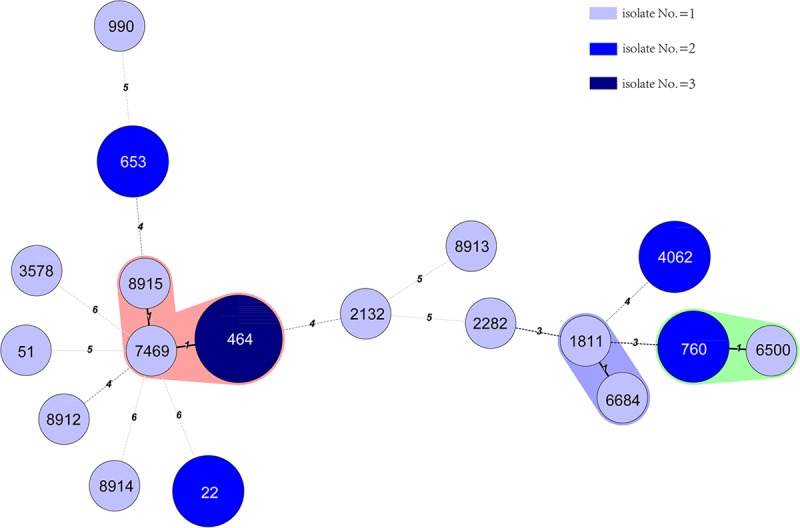
Phylogenetic relatedness of 24 *C. jejuni* isolates. The minimum spanning tree showing the relatedness of 24 *C. jejuni* strains, which was based on the STs using Bionumerics software, version 5.1. Different circles correspond to different STs. The color of the circles indicates the number of isolates belonging to each ST. There was only one different allele between ST1811 with 6684, ST760 with 6500, and ST8915, 7469 with 464. However, there was no epidemic relatedness between those cases that we could detect.

Eighteen STs were identified from the 24 *C. jejuni* isolates. Four novel STs were identified in this study; they were ST8912, 8913, 8914, and 8915 (The IDs of the isolates in PubMLST for the four new STs were 60894 to 60897). The frequently detected STs were ST464 (three isolates), ST22 (two isolates), ST760 (two isolates), ST653 (two isolates), and ST4062 (two isolates). The other 13 STs were identified for 13 single isolates. One *C. coli* isolates was ST5511, which belonged to the ST828 clonal complex and the other isolate belonged to ST2737. The minimum spanning tree for 24 *C. jejuni* isolates was constructed and shown in (**Figure 2**).

## Discussion

Acute diarrhea remains a serious public health problem in developed and developing countries (Lynch et al., 2011). Bacterial pathogens have been confirmed as the leading cause of acute diarrhea amongst outpatients in China (Wang et al., 2015; Shen et al., 2016; Chang et al., 2017). *Campylobacter* infection is considered to be one of the leading causes of bacterial gastroenteritis in the developed countries (Bresee et al., 2012; Wei et al., 2014; Kaakoush et al., 2015; Kumagai et al., 2015; Nohra et al., 2016; Pavlova et al., 2016). One comparable study reported no *Campylobacter* infections among diarrheal patients in a developed region neither in children (under 5 years, 0/1422)) nor adults (0/1047) (Wang et al., 2015). The prevalence of *Campylobacter* in the elderly (≥65 years) with acute diarrhea was 0.5% (31/5967) (Zhang et al., 2017). Another study in Wuhan (Southwest China) in children with diarrhea showed that prevalence of *Campylobacter* infection was 2.9% (11/381) (Zhu et al., 2016). Recently, a case-control study of diarrheal children in Shanghai indicated that the *Campylobacter* isolation ratio from the diarrheal child was 5.1% (35/680) (Chang et al., 2017). These reports of *Campylobacter* infection in diarrheal patients showed varying observations. All of the previous studies to date have used this selective method. With an increase in drug resistance amongst specimen micro-organisms, the effectiveness of the selective medium and the enrichment broth is reduced. Laboratory experience for *Campylobacter* isolation is more needed to obtain the right colony from the micro-organisms on the selective medium. This might be one of the reasons why there has been such variations in prevalence reported for diarrheal patients here. In addition, more studies of *Campylobacter* prevalence in children have been conducted (Zhu et al., 2016; Chang et al., 2017). The surveillance for the adults is rare. In this study, we conducted surveillance for the diarrheal patients (aged from 15 to 87 years) using the isolation kit based on the filtration method which had been evaluated in the laboratory before the study (unpublished data).

The prevalence (7.0%, 26/380) of *Campylobacter* in this study was higher than the reported in diarrheal children(2.9%, 11/381) in Wuhan China (Zhu et al., 2016). The dominant specie isolated from the diarrheal patients was still *C. jejuni* (92.3%, 24/26) which is consistent with another recent study (Chang et al., 2017). Although there was no significant peak incidence, *Campylobacter* was the most commonly isolated pathogen from October to March. The *Campylobacter* reports were recorded for almost every month of the year. However, non-typhoidal *Salmonella* and DEC were only reported from April to October (**Table 1**). Throughout the whole year of surveillance, there was a significant increase for *V. parahaemolyticus* between July and September, and the isolation ratio in August reached 44.7% (17/38). *V. parahaemolyticus* seemed as the dominant summer diarrheal pathogen here. The summer seafood stalls are very popular in Beijing, it might be one of the reason for the higher *V. parahaemolyticus* infection during this period.

*Salmonella* is usually reported as one of the dominant bacterial pathogens in diarrheal patients here (Wang et al., 2015; Zhang et al., 2017). In order to obtain the specific characteristics of the prevalence for *Campylobacter* in the diarrheal patients, in this study, we assessed the difference in clinical outcome and incidence in different age group between patients with *Campylobacter* and non-typhoidal *Salmonella.* There was no significant difference between the number of patients who had stomach ache, nausea and vomiting, feeling thirsty, fever and feeling weak. However, the number of patients with dehydration and *Campylobacter* infection was significantly higher than the number of the patients with *Salmonella*. This indicated that clinical syndrome due to *Campylobacter* is more severe than that precipitated by *Salmonella.* There was also no significant difference between the ages of patients infected with *Campylobacter* or *Salmonella* (**Table 2**). Although most of the patients with *Campylobacter* were male (19 of 26), there was no significant difference between the infection ratio by different gender.

Poultry and livestock are considered as the major reservoir for *Campylobacter* infection. Chicken meat is commonly considered to be the suspected food source for *Campylobacter* infection in humans (Strachan et al., 2013). In this study, except for *V. parahaemolyticus* in the seafood and related products (*P* = 0.005, χ^2^ = 12.923), there was no significant difference of the suspected food type for other pathogens infection. These findings indicate that cross-contamination might be a major route of transmission for *Campylobacter* infection in China. However, we cannot rule out recall bias in those who were questioned.

Two *C. coli* isolates were sensitive only to florfenicol and chloramphenicol. All *C. jejuni* isolates were sensitive to erythromycin, which is consistent with previous reports (Zhang et al., 2016). Eighty-three point three percent (20/24) isolates were multi-drug resistant and over 90% of isolates were resistant to tetracycline and quinolone. In this study, we found that 58.3% (14/24) of human isolates were resistant to florfenicol, which is only licensed for use in animals and 16.7% (4/24) of isolates were resistant to chloramphenicol. The observation of highly florfenicol-resistant *Campylobacter* requires further investigation since human infection with this antimicrobial strain is unexpected. The dominant resistance pattern was nalidixic acid, ciprofloxacin, florfenicol, tetracycline and telithromycin combined resistance (**Figure 1**).

From the minimum spanning tree constructed from the 18 STs of the 24 *C. jejuni* isolates (**Figure 2**), we did not find any clusters related to the reported cases. ST8915 (1 isolate), 464 (3 isolates) and 7469 (1 isolate), had only one different allele between them. But to our knowledge, there was no epidemic relationship between those cases. There was only one different allele between ST1811 and 6684 but we could not epidemiologically relate these cases either.

In summary, the prevalence and characteristics of *Campylobacter* in diarrheal patients in Shunyi district (north–east Beijing) were examined in this study. It was the first surveillance report of the *Campylobacter* isolation with filtration method. Our study indicates that *Campylobacter* infection might be seriously under-ascertained in the diarrheal patients in China. A standard method that is independent of laboratory experience is crucial for future surveillance systems.

## Author Contributions

YZ, SZ, and HJ were involved in the collection of samples. YgL and SZ collected the clinical data. YZ and YdL performed the enteropathogen detection. HL and YF performed the molecular sub-typing and antibiotic susceptibility tests. MH, HM, and SZ performed the data analysis. YgL and MZ designed this study, drafted and revised this manuscript.

## Conflict of Interest Statement

The authors declare that the research was conducted in the absence of any commercial or financial relationships that could be construed as a potential conflict of interest.

## Footnotes

1 http://pubmlst.org/campylobacter/info/primers.shtml

2 https://pubmlst.org/campylobacter/
