# Supplementary material for: Prevalence and Molecular Characterization of Campylobacter spp. Isolated from Patients with Diarrhea in Shunyi, Beijing
Source: Front Microbiol. 2018 Jan 26;9:52. doi: 10.3389/fmicb.2018.00052 (PMC5790792; doi:10.3389/fmicb.2018.00052)
Supplement: Supplementary file 1 [file Presentation_1.ZIP › 319484_Zhang_Supplementary file S1.docx]

**Supplementary file S1**

**Questionnaire for Stool Sample Collection**

**Case code:**

**Information provider:**

(1) Patient (2) Family members or insiders (relationship：     )

**1.General information**

**1.1 Patient name：**

**1.2 Gender**  (1) Male (2) Female

**If female, whether pregnant**(1) Yes, ______week(s) (2) No

**1.3 Age (year)**

**1.4 Occupation**

**1.4.1** Employee in the catering industry (1) Yes (2) No

**1.4.2** Food industry or food service (1) Yes (2) No

**1.4.2** Medical staff (1)Doctors (2)Nurses (3)Care workers (4)Laboratory staff (5)Administrative staff (6)Others______(please elaborate)

**1.4.3**  Other Occupation

(1)Famers (2)Migrant worker (3)Industry blue collar Migrant worker (4)Cadre (5)Teachers (6)Students (7)Children in the kindergarten (8)Children not in the kindergarten (9)Housework or unemployed (10)Fisherman (11)Herdsman (12)Business services (13)Others______(please elaborate)

**1.5 Current study or work place:**

**1.6 Address：**____Province _____City _______County (district)

_______Township(street) ______Village (building) ____Team( unit)

**1.7 Household registration：**____Province _____City _______County (district) _______Township(street) ______Village (building) ____Team( unit)

**1.8 Nationality：**(1) China    (2) Others

**1.9 ID card or passport number:**

**1.10 Phone number:**

**2．Clinical manifestations**

**2.1 Date of onset:**    yy   mm    dd

**2.2 Location of onset:**

(1) China: ____Province _____City _______County

(2) Outside of China：

(3) On the transport: □ Aircraft □ Train □ Ship□ Car□ Other

**2.3Clinical Symptoms:**

| **Clinical Symptoms** | **yes** | **no** | **Remarks** |
| --- | --- | --- | --- |
| **Fever** |  |  | Body temperature(highest): |
| **Nausea and Vomiting** |  |  |  |
| **Dehydration** |  |  |  |
| **Feeling Thirsty** |  |  |  |
| **Feeling Weak** |  |  |  |
| **Other symptoms / signs** |  |  | (please elaborate): |

Definition of dehydration: The physical sign include decreased skin elasticity, dry skin mucous membrane, accelerated or weakened pulse, collapsed superficial vein, frosty limbs, decreased urine volume, etc.

**2.4 Outpatient / emergency treatment**

**2.4.1Dateoftreatment:**

**2.4.2 Hospitals/departments:**

**2.4.3 Whether used antibiotics in 3 days**

If used, the name of the drugs:

**2.5 Hospitalization and treatment**

**2.5.1 Whether hospitalization:**

(1) Yes (2) No（go to the part 3）

**2.5.2 Date of admission:** year month date

**2.5.3 Admission hospital name:**

**2.5.4 Admission number:**

**2.5.5 Admission diagnosis:**

(1) Diarrhea cases (2) Fever cases (3) Other clinical diagnosis:

**2.5.6 Treatment:**

**2.5.6.1Drug treatment:** (1) Antibiotics (2) Hormones (3) Antiviral drugs (4) Others:

**2.5.6.2 Whether to stay in ICU:**

(1)Yes（Check in date: year month date）   (2)No

**3．Epidemiological information**

**3.1 The suspected contaminated food eaten in 5 days:**

(1).Fruit and vegetables (2).Meats and related products (3).Grain and related products (4).Eggs and related products (5).Milk and related products (6).Bean and related products (7).Aquatic and related products (8).Water, Beverages and Herbs (9).Unknown

**4．Specimen collection**

| **Collection time** | **Specimen Type** | **sample size (g)** |
| --- | --- | --- |
|  |  |  |
|  |  |  |

**Specimen Type:**

(1)Watery-stools (2)Loose-stools (3)Mucus-stools

(4) Bloody-stools (5) Other (please elaborate):

Investigation units:

Investigation period:

Signature:
